# Improved dehydrogenation performance of LiAlH_4_ doped with BaMnO_3_

**DOI:** 10.1016/j.heliyon.2024.e31190

**Published:** 2024-05-13

**Authors:** N.A. Sazelee, Sami-ullah Rather, A.M. Sinin, M. Ismail

**Affiliations:** a Energy Storage Research Group, Faculty of Ocean Engineering Technology, Universiti Malaysia Terengganu, 21030, Kuala Nerus, Terengganu, Malaysia; b Department of Chemical and Materials Engineering, King Abdulaziz University, P.O. Box, 80204, Jeddah, 21589, Saudi Arabia; c STEM Foundation Centre, Universiti Malaysia Terengganu, 21030, Kuala Nerus, Terengganu, Malaysia

**Keywords:** Solid-state hydrogen storage, LiAlH_4_, Metal oxide, Desorption kinetics

## Abstract

Due to its high gravimetric capacity of hydrogen (10.5 wt%), LiAlH_4_ has been regarded as a promising material for solid-state hydrogen storage material for onboard usage. However, high decomposition temperature, poor kinetics and irreversibility retard its application. To counter this problem, various weight percentages of BaMnO_3_ are introduced into the LiAlH_4_ system as an additive in this work. As a result, the starting hydrogen release of LiAlH_4_ was reduced to 109−115 °C and the second desorption temperature occurred at around 134−158 °C, much lower than pure LiAlH_4_. The isothermal desorption kinetics also proved that faster desorption kinetics can be observed at 90 °C for 80 min. About 2.00−2.60 wt% of H_2_ could be desorbed by the composite, whereas only <1.00 wt% of H_2_ was desorbed by undoped LiAlH_4_. Additionally, adding BaMnO_3_ reduced the activation energies by 30 kJ/mol for the first stages and 34 kJ/mol for the second stages. Based on the X-ray diffraction result, the active species formed of MnO_2_ and Ba or Ba−containing materials are believed to be responsible for the noticeable enhancement in the desorption properties of LiAlH_4_.

## Highlights

• H_2_ storage properties of BaMnO_3_-doped LiAlH_4_ are investigated.
• Doped with BaMnO_3_ improved the H_2_ desorption properties of LiAlH_4_.
• The activation energy for two stages of the doped sample is decrease.
• MnO_2_ and Ba-containing species display a synergistic effect on the dehydrogenation of LiAlH_4_.

## 1 Introduction

Owing to its characteristics of having no carbon emissions (as the only product is water), high energy density and abundant resource, hydrogen has gained a lot of interest as the new green energy carrier [1,2]. However, the pervasive use of hydrogen as an energy carrier has been hampered by the lack of reliable and effective hydrogen transportation and storage technology [3,4]. The main goal of hydrogen storage is to ensure that it is always effective, useable at all places and times and secure. There are three methods to store hydrogen which are compressed gas [5,6], cryogenic liquid [7,8] and solid-state storage [9,10]. The scientific community is focusing more attention on solid-state hydrogen storage technology as the most secure and dependable way to store hydrogen under optimal working conditions for various kinds of purposes [[11], [12], [13], [14], [15]]. Furthermore, the solid-state method allows a significant amount of hydrogen to be stored in a relatively small space [16,17]. Aluminium hydride (AlH_3_) has been considered as a possible hydrogen storage material because of its high hydrogen storage capacity [18]. Unfortunately, after desorption, AlH_3_ is converted to pure Al with poor reversibility, which makes it impractical to use as a hydrogen energy carrier [19]. LiAlH_4_ has been gaining a lot of interest as an ideal solid-state hydrogen storage material because of its high energy content (10.5 wt%) [20,21]. The thermal dissociation of LiAlH_4_ proceeds in a few steps below [22].

(1) 3LiAlH_4_ → Li_3_AlH_6_ + 2Al + 3H_2_

(2) Li_3_AlH_6_ → 3LiH + Al + 3/2H_2_

(3) 3LiH + 3Al → 3LiAl + 3/2H_2_

However, LiAlH_4_ suffers from sluggish absorption and desorption kinetics and required high temperatures to release hydrogen (at the third decomposition stage). The absorption of LiAlH_4_ is possible under ultra-high pressure [23,24]. Numerous attempts have been put forward to promote the kinetics and boost the hydrogen desorption of LiAlH_4_ including adding additives/catalysts and a milling process [[25], [26], [27], [28], [29]]. In addition, Zhang and co-workers [30] exposed that some additives could exhibited unique morphology which may affected the hydrogen storage performance of LiAlH_4_.

In general, it has been proven that metal oxide additives are considered to be the most beneficial additives for improving the desorption properties of LiAlH_4_. Ali et al. [31] evaluated the role of CoTiO_3_ to boost the desorption properties of LiAlH_4_. The research findings disclosed that the first two stages of the starting hydrogen release reduced from 145 °C to 115 °C and 175 °C to 145 °C, respectively. In light of the findings, the active species of AlTi and Co or Co−containing during the heating process were responsible for a significant enhancement of the desorption properties of LiAlH_4_. Li and colleagues [32] confirmed the initial desorption of LiAlH_4_ had dropped to 94 °C for the first stage and 72 °C for the second stage after the presence of NiFe_2_O_4_. As confirmed by the XRD analysis, the desorption properties of LiAlH_4_ get enhanced by the *in situ* formation of LiAlO_3_, LiFeO_2_ and Al–Ni during the heating process.

Ismail et al. [33] looked into the valuable impact of Al_2_TiO_5_ on the desorption properties of LiAlH_4_. It is captivating to point out that small addition of Al_2_TiO_5_ can desorb up to 3.5 wt% of H_2_ in 1 h and start to release hydrogen at 90 °C and 137 °C for the first two stages, respectively. For instance, Wei et al. [34] selected spinel ferrite nanoparticles such as NiFe_2_O_4_, CoFe_2_O_4_, MnFe_2_O_4_ and CuFe_2_O_4_ as an additive to be introduced into LiAlH_4_. This additive was prepared using the thermal decomposition method. The results show that the desorption performance of LiAlH_4_ doped with this spinel ferrite reduced in the range of 69 °C–100 °C for the first stages and 129 °C–147 °C for the second stages. This is much lower compared with pristine LiAlH_4_. Additional studies have demonstrated that the active species of LiFeO_2_ serve as active sites, thus improving the desorption of LiAlH_4_.

In the context of the metal oxides mentioned earlier, another metal oxide (BaMnO_3_) was used to improve the kinetics of LiAlH_4_ desorption. In particular, a previous study demonstrated that the introduction of 10 wt% BaFe_12_O_19_ could effectively enhance the hydrogen desorption properties of LiAlH_4_ [35]. Faster desorption kinetics of BaFe_12_O_19_ doped LiAlH_4_ can be observed compared to undoped LiAlH_4_. BaFe_12_O_19_ doped LiAlH_4_ can desorb ∼4.1 wt% of H_2_ as opposed to undoped that only desorbed less than 1.0 wt% of H_2_ under the same circumstances. Other catalysts like Mn also have been developed and their catalytic effect on the hydrogen storage of LiAlH_4_ has been studied. For example, Wei et al. [34] found out that the addition of MnFe_2_O_4_ led to a 76.4 °C reduction in desorption temperature compared with pure LiAlH_4_. Moreover, as reported by Zhai et al. [36], smaller particle sizes were observed after being doped with MnFe_2_O_4_ which activates the surfaces of the samples and increases their surface area resulting in more hydrogen can be released. In addition, the activation energies proved remarkably lowered by 44.9 kJ/mol and 104.9 kJ/mol for the first two stages indicating a significant improvement by doping with MnFe_2_O_4_. This enhancement could be clarified through the formation of Fe–O and Mn or Mn−containing during the heating process.

Therefore, in this study, BaMnO_3_ was used as an additive for enhancing the desorption kinetics of LiAlH_4_. Moreover, the role of the active species was also discussed. Additionally, the microstructures of the samples were also evaluated to give a better understanding. As far as we know, no studies have been reported on the performance of LiAlH_4_ doped with BaMnO_3_.

## 2 Methodology

In this study, BaMnO_3_ was used as an additive and was synthesized by using the solid-state method as discussed in a previous study [37]. BaMnO_3_ was initially mixed with LiAlH_4_ (≥99 %) obtained from Sigma Aldrich in a weight ratio of 40:1 using planetary ball milling (NQM−0.4) for 1 h at 400 rpm. In this study, the preparation was done in an MBRAUN UNIlab argon-filled glove box with oxygen and moisture levels below 1 ppm due to the high reactive properties of the raw materials with moisture and oxygen.

Temperature-programmed desorption (TPD) and the desorption kinetics were accomplished by using Advanced Materials Corporation. The TPD curves were carried on at rates of 5 °C/min from ambient temperature to 250 °C. The desorption kinetics was measured under 1.0 atm pressure at 90 °C. The lowest starting point of hydrogen release and the amount of hydrogen desorbed were evaluated. Approximately, 150 mg of the samples was loaded into a sample vessel before it was connected to the PCT instrument. For this step, the instrument is connected to a computer and controlled by software (GrcLV), which performs in a fully automatic operation. On a Mettler Toledo DSC/TGA 1, differential scanning calorimetry (DSC) analysis of the sample was completed. In an alumina crucible, 3–5 mg of samples has been added and different heating rates (15, 20, 25 and 30 °C/min) were used to heat the samples to 300 °C in a 50 mL argon atmosphere. The alumina crucible was then sealed in a glass bottle to avoid oxidation of the samples during the transfer from the glove box to the DSC apparatus.

The X-ray diffraction (XRD) pattern has been used to characterize the structure of the samples with Cu−Kα radiation from 20° to 80° with a speed of 2.00°/min. To minimize the oxidation of the samples, a small amount of the samples was spread uniformly on the sample holder and covered with scotch tape and followed by sealing with plastic wrap. The morphology was studied by scanning electron microscope (SEM) (JEOL JSM-6360LA). As an attempt to avoid the oxidation and moisture affection of the samples, the samples were uniformly spread on a carbon tape and was coated with ultrathin gold spray using vacuum sputter coating. The vibration of the samples and Al–H stretching and bending was measured by using Fourier transform infrared spectroscopy (FTIR) (Tracer-100 Shimadzu). The samples characterization was conducted in the mode of attenuated total reflectance (ATR) equipped recorded from 40 scans between 400 and 2000 cm^−1^.

## 3 Result and discussions

**Fig. 1:**
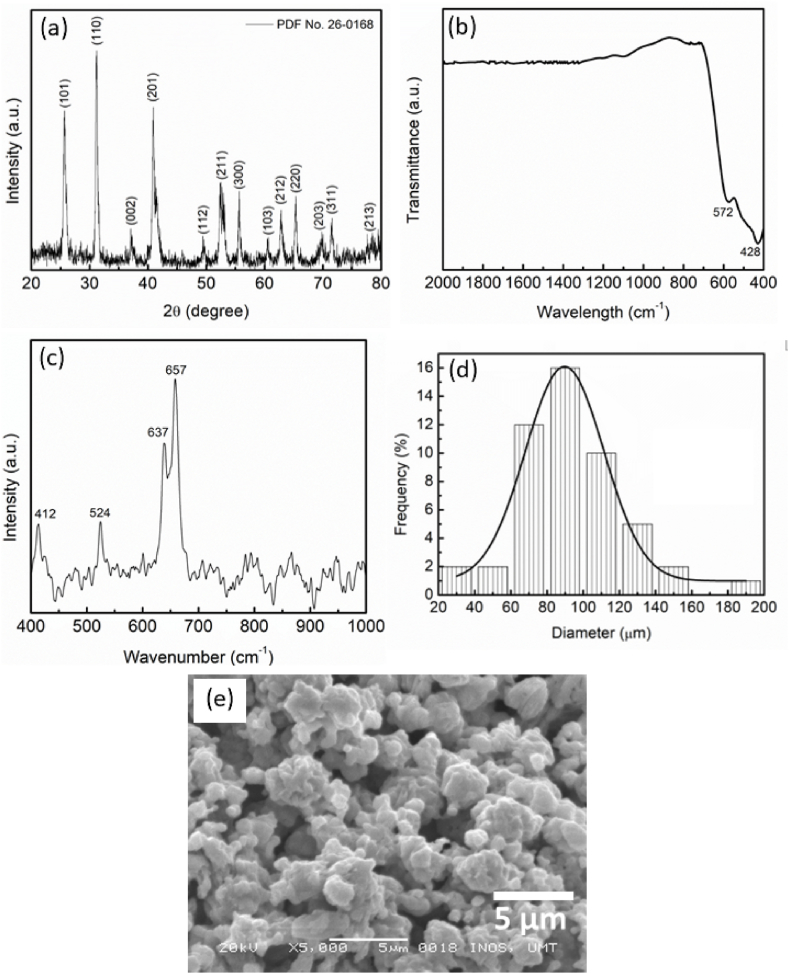
(a) XRD pattern, (b) FTIR spectra, (c) Raman spectra, (d) PSD and (e) SEM images of hexagonal BaMnO_3_ [37].

The BaMnO_3_ additives were characterized by using XRD, FTIR, Raman spectroscopy and SEM in order to evaluated the purity and morphology of BaMnO_3_. As shown in Fig. 1(a), there was no other phase observed, indicating that this BaMnO_3_ was completely pure. The diffraction peaks at 2θ of 25.79°, 31.36°, 36.37°, 41.05°, 49.47°, 52.54°, 55.80°, 60.57°, 62.94°, 65.42°, 69.76°, 71.55° and 78.50° correspond to (101), (110), (002), (201), (112), (211), (300), (103), (212), (220), (203), (311) and (213), respectively. The FTIR spectra showed the characteristic peaks related to Ba–O at 428 cm^−1^ and Mn–O at 572 cm^−1^ as revealed in Fig. 1(b)–as mentioned by Razavi et al. [38] and Gholamrezaei et al. [39]. Furthermore, the Raman spectra in Fig. 1(c) below presented the typical peaks of BaMnO_3_, which are at 412, 524, 637 and 657 cm^−1^. This was also confirmed by the Raman spectra of hexagonal BaMnO_3_ as investigated by Roy and Budhani [40]. The results of XRD, FTIR and Raman spectroscopy showed that BaMnO_3_ was successfully synthesized using the solid-state method without any impurities. Meanwhile, Fig. 1(d) shows the particle size distribution (PSD) of BaMnO_3_ evaluated using ImageJ. It can be seen that the ImageJ measurement showed that the average particle size of BaMnO_3_ was 90 μm. BaMnO_3_ has an agglomeration shape, as shown in the SEM images in Fig. 1(e).

**Fig. 2:**
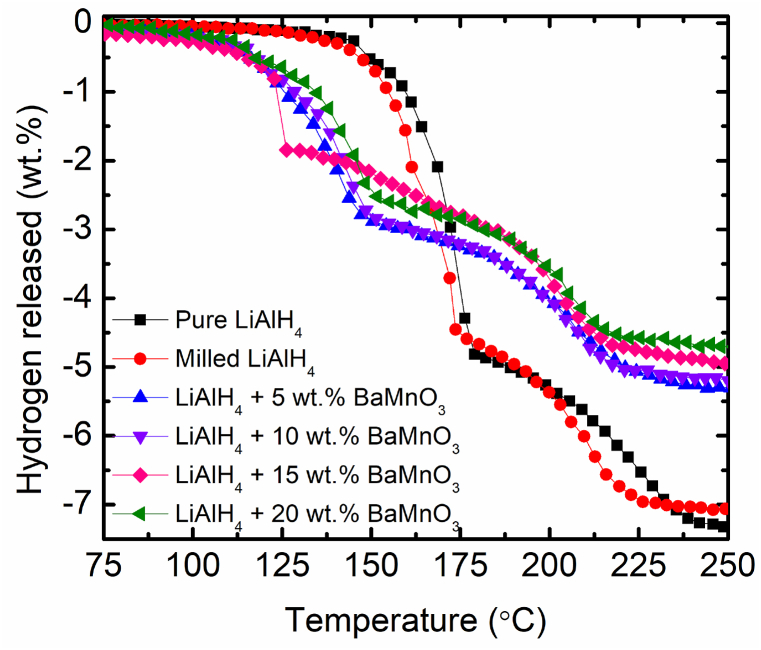
TPD curves of pure LiAlH_4_, milled LiAlH_4_ and LiAlH_4_ doped with xwt.% of BaMnO_3_ (x = 5, 10, 15 and 20).

Fig. 2 illustrates the TPD curves of pure LiAlH_4_, milled LiAlH_4_ and LiAlH_4_ doped with various percentages of BaMnO_3_. The desorption curves made it obvious that the inclusion of BaMnO_3_ drastically reduced the onset desorption temperature of LiAlH_4_ for both stages. For pure LiAlH_4_, it started to release at 151 °C and the second step occurs at 184 °C with 7.31 wt% of hydrogen release. Due to the milling process, milled LiAlH_4_ experiences a small reduction in onset desorption temperature (4 °C for the first step and 8 °C for the second) compared with pure LiAlH_4_. A study reported by Zhai and co-workers [36] exposed that starting of hydrogen release lowered from 150 °C to 132.5 °C for the first stages and 190 °C to 172.5 °C for the second stages after the milling process. Smaller particles size of the samples created by the milling process will shorten the diffusion length of LiAlH_4_, thus reducing the starting point of hydrogen release [41].

The starting point of hydrogen release is even lower after the inclusion of BaMnO_3_. After 5 wt% of BaMnO_3_ was added, the process occurs at 110 °C and 155 °C for the first two stages, respectively. The start of hydrogen released is reduced to 109 °C and 158 °C for the first two stages, respectively when the additive amount is increased to 10 wt%. During the process, the sample with 5 wt% BaMnO_3_ release about 5.34 wt% of H_2_ whereas 5.26 wt% of H_2_ for 10 wt% of BaMnO_3_. With an H_2_ release rate of 5.26 wt%, 15 wt% of BaMnO_3_ has an onset desorption temperature at 109 °C and 134 °C for the first two stages, respectively. The onset desorption temperature drops to 115 °C and 158 °C with a 4.70 wt% total H_2_ release as the amount of BaMnO_3_ rises to 20 wt%. The decreasing of total hydrogen release compare with the undoped sample are due to the dead weight of the BaMnO_3_ since no hydrogen is in such a species. According to a prior study by An et al. [42], the 0.8 wt% loss is caused by the dead weight after Ni@C was added since no hydrogen in such a species. From the result obtained, the starting of hydrogen released by LiAlH_4_ was lowered by adding BaMnO_3_ as an additive.

**Fig. 3:**
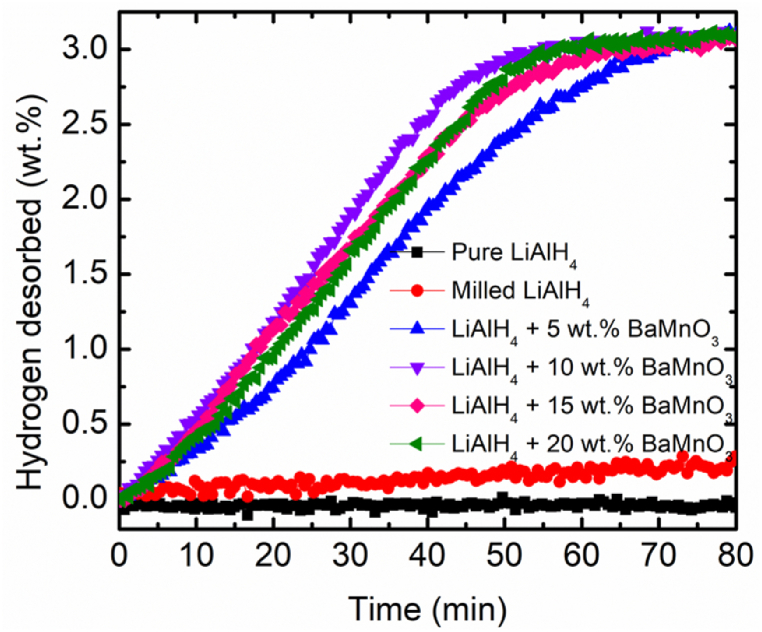
Isothermal desorption kinetics of pure LiAlH_4_, milled LiAlH_4_ and LiAlH_4_ doped with xwt.% of BaMnO_3_ (x = 5, 10, 15 and 20).

Fig. 3 shows the relations of time and amount of hydrogen desorbed of the pure LiAlH_4_, milled LiAlH_4_ and LiAlH_4_ doped with 5, 10, 15 and 20 wt% of BaMnO_3_. Within 80 min at 90 °C, pure LiAlH_4_ and milled LiAlH_4_ desorbed approximately less than 1.00 wt% of H_2_. However, a faster desorption rate of H_2_ can be observed after adding with BaMnO_3_ additive. The samples doped with 10 wt% of BaMnO_3_ present the largest amount of H_2_ desorbed (2.56 wt%) within 40 min. Comparatively, the samples doped with 5 wt% of BaMnO_3_ can desorb only 2.02 wt% of H_2_ under the same conditions. Besides, the same amount of hydrogen desorbed (2.24 wt% of H_2_) can be seen for the samples doped with 15 and 20 wt% of BaMnO_3_ under the same circumstances. A previous study by Li and co-workers [43] exposed that the addition of metal oxide (CoFe_2_O_4_) exhibits superior catalytic performance and significant enhancement for the desorption kinetics of LiAlH_4_. Even at the lowest temperature (90 °C), 2 mol% CoFe_2_O_4_ + LiAlH_4_ can desorbed ∼1.5 wt% of H_2_ within 25 min. For the curve of pure LiAlH_4_, sluggish desorption kinetics could be observed even if the sample was heated at the same temperature within 180 min. Therefore, it can be inferred that adding BaMnO_3_ improves the desorption kinetics and 10 wt% of BaMnO_3_ occupied the ideal desorption kinetics behaviour and was selected for another characterization.

**Fig. 4:**
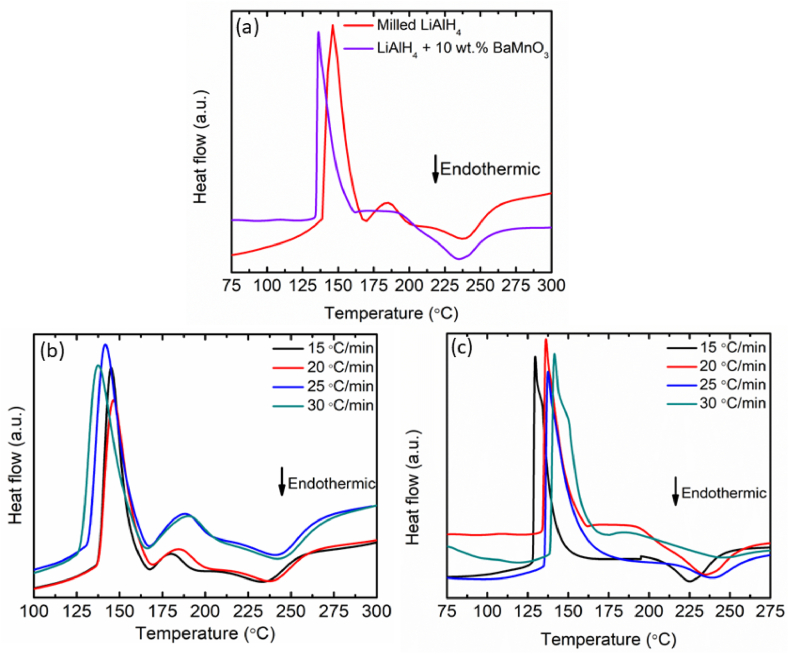
DSC curves of (a) milled LiAlH_4_ and LiAlH_4_ doped with 10 wt% of BaMnO_3_ at 20 °C/min, (b) milled LiAlH_4_ and (c) LiAlH_4_ doped with 10 wt% of BaMnO_3_ at various heating rates.

The DSC curves of milled LiAlH_4_ and LiAlH_4_ doped with 10 wt% BaMnO_3_ at 20 °C/min are presented in Fig. 4(a) below. The DSC curves of the milled LiAlH_4_ show 4 peaks (2 endothermic and 2 exothermic) which are consistent with the earlier report from 25 to 300 °C. The presence of surface hydroxyl impurities in the LiAlH_4_ at 145 °C leads the 1st exothermic peak to appear. The melting point of LiAlH_4_ is labelled as the 1st endothermic peak at 169 °C. The desorption of LiAlH_4_ is assigned to the 2nd exothermic peak at 185 °C while the desorption of Li_3_AlH_6_ occurs at 241 °C which corresponds to the 2nd endothermic peak. In contrast, adding 10 wt% of BaMnO_3_ reduced the peak from 4 to 2. The exothermic and endothermic peaks centered at 134 °C and 233 °C correspond to the desorption peak of LiAlH_4_ to Li_3_AlH_6_ and the desorption peak of Li_3_AlH_6_ to LiH, respectively. The addition of BaMnO_3_ as an additive significantly moved the desorption peak to a lower temperature. The DSC curves of milled LiAlH_4_ and LiAlH_4_ doped with 10 wt% of BaMnO_3_ have been displayed in Fig. 4(b) and (c) below at various heating rates, respectively. With an increase in heating rate, the peak temperature can be seen shifting toward the side with higher temperatures. As revealed by Ismail and co-workers [44], the number of peaks decreased to 2 after the addition of SrTiO_3_ to LiAlH_4_ and occurred at a lowered temperature than the milled LiAlH_4_. This proved that adding SrTiO_3_ has a significant impact on LiAlH_4_ performance. Hence, in this research, the synergistic effect of BaMnO_3_ towards the decomposition of LiAlH_4_ in the DSC analysis has been proven.

**Fig. 5:**
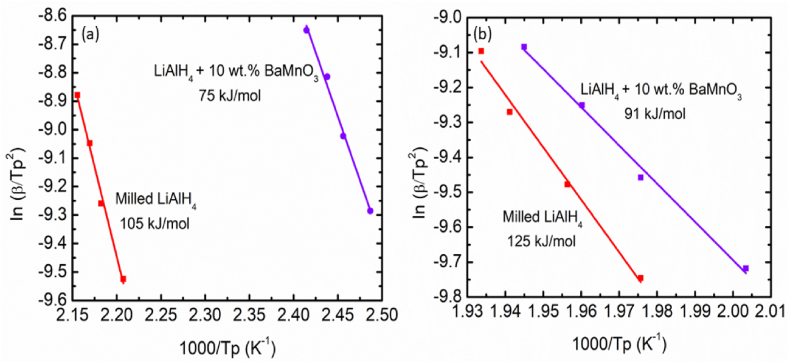
Activation energies for milled LiAlH_4_ and LiAlH_4_ doped with 10 wt% of BaMnO_3_ at (a) the first stages and (b) the second stages.

Using the Kissinger equation below, the activation energies (*E*_*A*_) can be calculated from the data obtained by the DSC measurements at various heating rates for the milled LiAlH_4_ and LiAlH_4_ doped with 10 wt% of BaMnO_3_:

(4) ln [*β*/*T*_*p*_^2^] = -*E*_*A*_ / R*T*_*p*_ + A

where *T*_*p*_ = peak temperature in the DSC curve, *β* = heating rate of the samples, R = gas constant and A = linear constant. By fitting the data points, the apparent activation energies for both samples were displayed in Fig. 5(a) and (b). The activation energies of the LiAlH_4_ doped with 10 wt% of BaMnO_3_ were evaluated to be 75 kJ/mol for the 1st stages and 91 kJ/mol for 2nd stages, respectively. Meanwhile, the activation energies for milled LiAlH_4_ were 105 kJ/mol for the 1st stage and 125 kJ/mol for the 2nd stage. These values are 29 % and 27 % less than milled LiAlH_4_. The LiAlH_4_ system after adding K_2_NbF_7_ also showed the same phenomenon [45]. Adding K_2_NbF_7_ reduced the activation energies by 24 kJ/mol and 26 kJ/mol for the first two stages, respectively. Their result proved that the addition of additives can lower the activation energies for both stages. Thus, it can be hypothesized that the addition of BaMnO_3_ additive was beneficial for LiAlH_4_, thus helping to exert its better catalytic action.

**Fig. 6:**
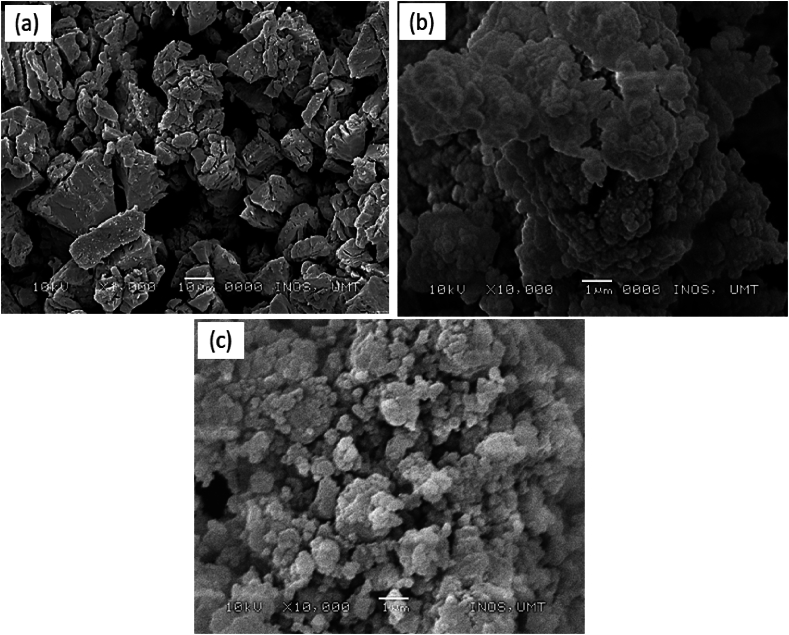
Morphology of (a) pure LiAlH_4_, (b) milled LiAlH_4_ and (c) LiAlH_4_ doped with 10 wt% of BaMnO_3_.

The morphology of pure LiAlH_4_, milled LiAlH_4_ and LiAlH_4_ doped with 10 wt% BaMnO_3_ were characterized by SEM as displayed in Fig. 6 below. As revealed in Fig. 6(a), LiAlH_4_ has irregular block shape particles. The milling process for 1 h resulted in smaller and agglomerated particle sizes as depicted in Fig. 6(b). As pointed out earlier, the milling method might boost the desorption properties of LiAlH_4_. The reduction in particle size produces via the milling process results in a more active surface of the materials. Besides, more defects are created inside the samples, which promotes the H atom to release more hydrogen [46]. Particularly, when used in a ball mill process, the development of a new interface under a condition of high-impact stress provide an effective pathway for hydrogen atom as reported by a previous study [47]. However, LiAlH_4_ cannot be used commercially with just ball milling operations. The desorption properties of LiAlH_4_ may be enhanced even more after the inclusion of the BaMnO_3_ additive. The morphology of LiAlH_4_ doped with 10 wt% of BaMnO_3_ is presented in Fig. 6(c). As proven in the figure below, the particle size becomes less aggregate and smaller compared with milled LiAlH_4_. This outcome is in line with the results of numerous studies including by Yahya and co-workers [48] who discovered that adding a catalyst could decrease the particle size of the hydrogen storage materials and prevent the sample from clumping together. Sun et al. [49] indicated that mixing LiAlH_4_ with NiCl_2_ together can obtain finer particles. Finer the size of the particles also influenced the desorption kinetics of the samples since the diffusion path of the samples decreased, hence more hydrogen has been released [50]. Materials with smaller grain sizes have more boundaries which allow the hydrogen to diffuse faster within the materials [51]. Therefore, the SEM image provides a piece of evidence that the BaMnO_3_ additive is essential for accelerating solid-state hydrogen storage materials of LiAlH_4_.

**Fig. 7:**
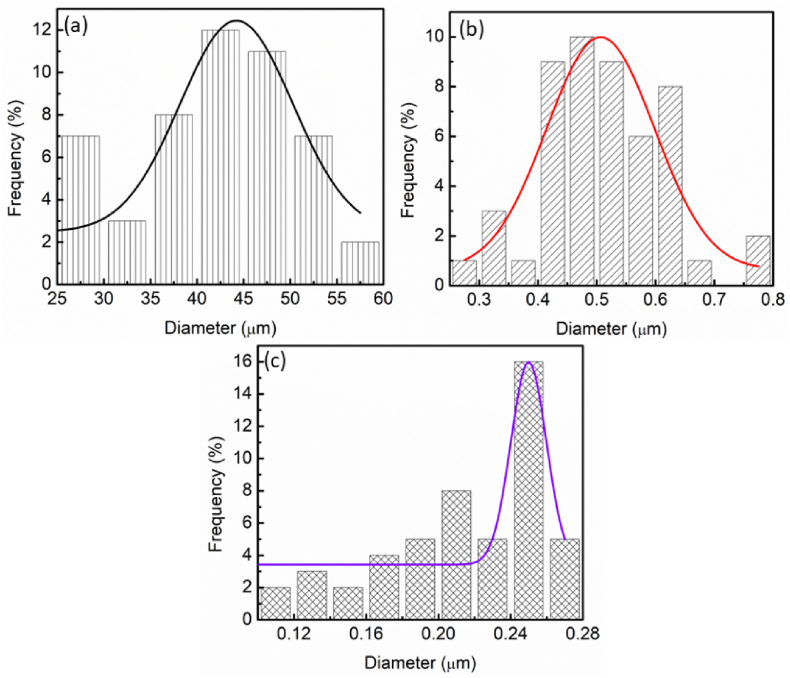
PSD of (a) pure LiAlH_4_, (b) milled LiAlH_4_ and (c) LiAlH_4_ doped with 10 wt% of BaMnO_3_.

The PSD of each sample was analyzed by using ImageJ. For pure LiAlH_4_, the particle size distribution was 44 μm as shown in Fig. 7(a). The PSD declined to 0.5 μm after 1 h milling of LiAlH_4_ as illustrated in Fig. 7(b). A significant particle size reduction is observed after the samples were milled for 5 h as stated by Maddah et al. [52]. The same phenomenon was also observed in a previous study [53]. The average particle size of MgH_2_ was lowered from 30 μm to 2.2 μm after 5 h of the milling process. As proven by this, the size of the samples was influenced by the milling process. In addition, the mean particle of the sample also decreases obviously after the milling process from 70 μm to 40 μm as exposed by Li et al. [54]. As discussed in their study, decreasing the particle size will increase the specific surface area of the sample and enhance the performance of hydrogen storage performance of TiFe alloy. However, after the presence of 10 wt% of BaMnO_3_ (Fig. 7(c)), the particle distribution of the composite reduced to 0.25 μm. This proved that the particle size becomes smaller compared with milling LiAlH_4_. Smaller particle sizes obtained by this composite form a larger surface area and more grain boundaries to promote the desorption process of LiAlH_4_. A similar outcome also has been revealed by Zhang and colleagues [30].

**Fig. 8:**
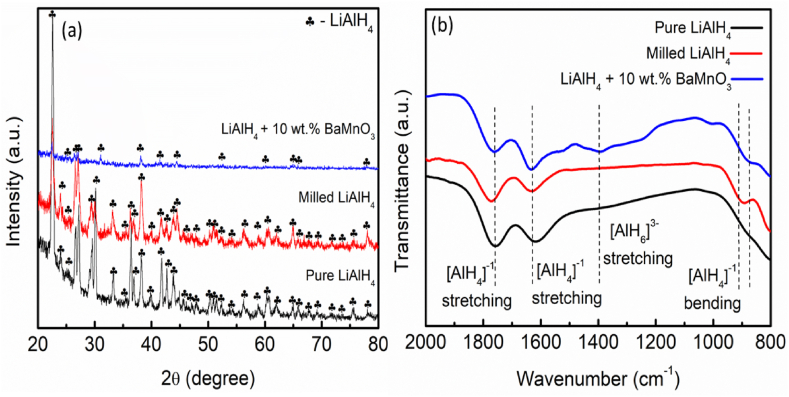
(a) XRD pattern and (b) FTIR spectra of pure LiAlH_4_, milled LiAlH_4_ and LiAlH_4_ doped with 10 wt% of BaMnO_3_.

Fig. 8(a) and (b) present the XRD pattern and FTIR spectra of the pure LiAlH_4_, milled LiAlH_4_ and LiAlH_4_ doped with 10 wt% BaMnO_3_, respectively. Fig. 8(a) reveals that pure LiAlH_4_ can be observed as it matches with JCPDS card no. 73–461. Milling the sample for 1 h decreases the intensity of the sample. No other new peaks were detected during the milling process which indicated that the sample remains stable. Besides, adding 10 wt% of BaMnO_3_ revealed that no newly formed phases were detected. No peaks of BaMnO_3_ were detected due to the low amount of the additive or the additive being amorphous. In order to confirm the slight decomposition of LiAlH_4_ that occurred after milling with BaMnO_3_, the samples were evaluated using FTIR (Fig. 8(b)). Fig. 8(b) also includes the as-received and as-milled LiAlH_4_ samples for comparison. As presented in Fig. 8(b), the vibration of the Al–H bond at 1401 cm^−1^ for the BaMnO_3_-doped LiAlH_4_ sample strongly supports the presence of Li_3_AlH_6_ (Al–H stretching mode), which cannot be detected in the XRD pattern. It can be concluded that the addition of BaMnO_3_ allows that LiAlH_4_ decomposes to Li_3_AlH_6_ and Al, as in Eq. (1). The bands for the LiAlH_4_ doped with 10 wt% BaMnO_3_ remain unchanged in position but the intensity has been reduced compared with pure LiAlH_4_. Two regions of Al–H modes were discovered (i) [AlH_4_]^−^ bending at 800−900 cm^−1^ and (ii) [AlH_4_]^−^ stretching at 1600−1800 cm^−1^. A similar study was disclosed by Yusnizam et al. [55] which observed that a new peak at 1403 cm^−1^ was detected which shows that the addition of TiSiO_4_ could weak the Al–H bond of LiAlH_4_.

**Fig. 9:**
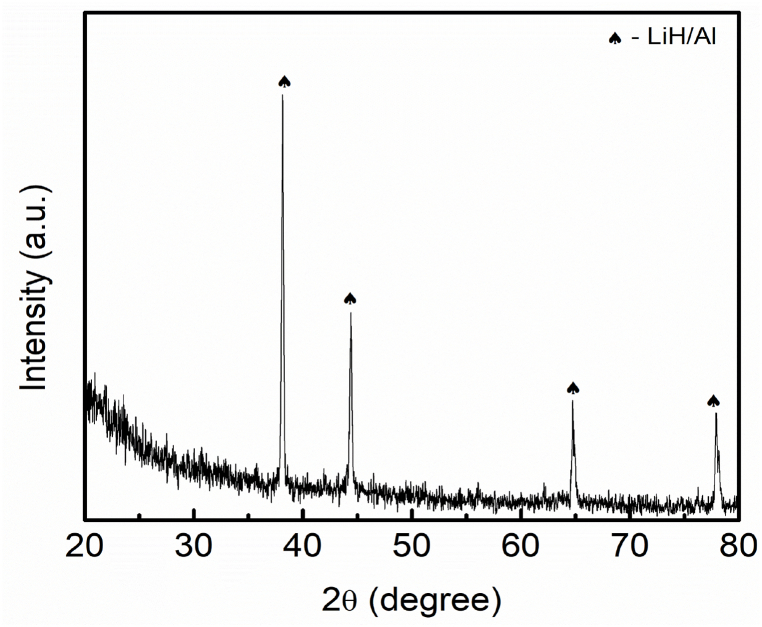
XRD pattern of the desorption LiAlH_4_ doped with 10 wt% of BaMnO_3_ composites at 250 °C.

An intriguing aspect of the LiAlH_4_ doped with 10 wt% BaMnO_3_ is elucidated by the XRD pattern displayed in Fig. 9. At 250 °C, the peaks of LiH/Al were detected as the main products. Nevertheless, no peaks of LiAlH_4_ and Li_3_AlH_6_ were detected indicating that LiAlH_4_ was completely decomposed into LiH and Al. However, no peaks of Ba or Mn were detected in this reaction.

**Fig. 10:**
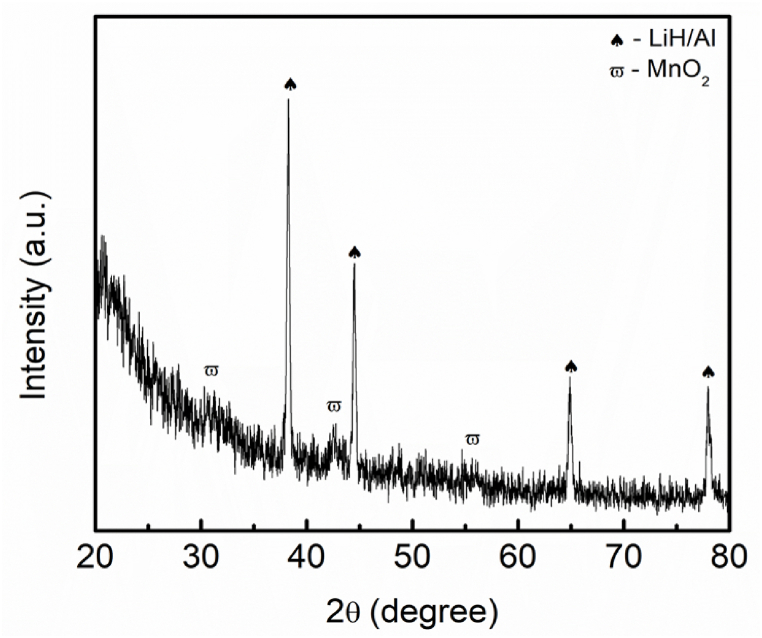
XRD pattern of the desorption LiAlH_4_ doped with 30 wt% of BaMnO_3_ composites at 250 °C.

For the sake of understanding the mechanism reaction between LiAlH_4_ and BaMnO_3_, the amount of BaMnO_3_ was increased to 30 wt%. New peaks of MnO_2_ were detected in this reaction as shown in Fig. 10. For their part, Wan et al. [56] revealed Mn or Mn−containing species formed between the reaction of sodium alanate and MnFe_2_O_4_ enhanced the desorption properties of sodium alanate. It is described that the starting point of hydrogen release of sodium alanate was lowered by 84 °C, 88 °C and 84 °C for all stages, respectively. Besides, Naik and colleagues [57] exposed the superior effect of Mn on enhancing the desorption performance of NaAlH_4_. Wan et al. [58] clarified that the performance of MgH_2_–LiAlH_4_ enhanced after the inclusion of MnFe_2_O_4_. Low desorption temperature and faster absorption and desorption kinetics can be observed which are primarily responsible for the formation of Mn−containing and Fe_0·872_O phases during the heating process. The Mn peaks still cannot be spotted relatively due to the peak being in an amorphous state or lower amount. Additionally, in this study, the peak of Ba or Ba−containing still cannot be found due to an amorphous phase. As indicated by a previous study [35], the active species of Ba or Ba−containing between the reaction of LiAlH_4_ and BaFe_12_O_19_ could reduce the starting of hydrogen release by 51 °C and 24 °C and reduced the activation energies for 32 kJ/mol and 22 kJ/mol for the first two stages of LiAlH_4_, respectively. In addition, the Ba or Ba−containing also could not be detected in the XRD during the heating process of MgH_2_ and BaFe_12_O_19_ even though the catalyst was increased to 20 wt% of BaFe_12_O_19_ [59]. Besides, the Ba or Ba−containing that formed acts as a real catalyst which boosts the performance of hydrogen storage of MgH_2_. Wang et al. [60] in their research indicated that the addition of BaTiO_3_. Interestingly, BaTiO_3_ remains stable after milling, after the desorption and absorption process which indicates that there is no reaction between MgH_2_ and BaTiO_3_. However, BaTiO_3_ plays a significant role during the absorption and desorption process of MgH_2_ which results in a decrement in the starting of hydrogen release and faster absorption and desorption kinetics.

## 4 Conclusion

The positive effect of adding metal oxide additives through the milling process on the desorption properties of LiAlH_4_ has been recognized by many researchers. In this study, 5, 10, 15 and 20 wt% of BaMnO_3_ were mixed with LiAlH_4_ by the milling method. It is revealed that BaMnO_3_ could highly boost the desorption properties of LiAlH_4_. Specifically, LiAlH_4_ doped with xwt.% of BaMnO_3_ (where x = 5, 10, 15 and 20) composite remarkably reduced the onset operating temperature to 109−115 °C for the first stage and 134−158 °C for the second stage, much lower than that of milled LiAlH_4_ (147 °C and 176 °C) and pure LiAlH_4_ (151 °C and 184 °C). The desorption kinetics of LiAlH_4_ was significantly faster compared with milled LiAlH_4_ and pure LiAlH_4_. Approximately 2.00–2.60 wt% of H_2_ can be desorbed by xwt.% of BaMnO_3_ (where x = 5, 10, 15 and 20) doped with LiAlH_4_ within 80 min at 90 °C compared with milled LiAlH_4_ and pure LiAlH_4_ (<1.00 wt%). The activation energies of the composites were lowered to 75 kJ/mol and 91 kJ/mol and this is particularly lower than milled LiAlH_4_. From the SEM images, the enhancement in the desorption performance of LiAlH_4_ is also attributed to the smaller particle size and less aggregate which produce more grain boundaries and high surface defect. From the FTIR result, the results also demonstrated that for the LiAlH_4_ doped with 10 wt% of BaMnO_3_, BaMnO_3_ additives can weaken the LiAlH_4_ bonding strength, thus leading to the lower desorption temperature of LiAlH_4_. The formation of Ba and Ba−containing and MnO_2_ after the heating process are believed to be the active species that helped to enhance hydrogen storage performance of the composites. Thus, it is concluded that the BaMnO_3_ is a good additive in improving the hydrogen desorption kinetics of LiAlH_4_.

## Data availability statement

We have not deposited the data associated with this study in a publicly accessible repository because it relates to our future work. However, we will make the data available upon request.

## CRediT authorship contribution statement

**N.A. Sazelee:** Writing – review & editing, Writing – original draft, Methodology, Investigation, Formal analysis, Data curation. **Sami-ullah Rather:** Writing – review & editing, Validation, Resources, Data curation. **A.M. Sinin:** Writing – review & editing, Formal analysis. **M. Ismail:** Writing – review & editing, Writing – original draft, Validation, Supervision, Resources, Project administration, Methodology, Investigation, Funding acquisition, Formal analysis, Data curation.

## Declaration of competing interest

The authors declare that they have no known competing financial interests or personal relationships that could have appeared to influence the work reported in this paper.
